# Supplementary material for: Flowers of Astragalus membranaceus var. mongholicus as a Novel High Potential By-Product: Phytochemical Characterization and Antioxidant Activity
Source: Molecules. 2019 Jan 25;24(3):434. doi: 10.3390/molecules24030434 (PMC6384981; doi:10.3390/molecules24030434)
Supplement: Supplementary file 1 [file molecules-24-00434-s001.pdf]

Supplementary Material

Flowers of *Astragalus membranaceus* var. *mongholicus* as a novel high potential by-product: Phytochemical characterization and antioxidant activity

Yuan Li [1], Sheng Guo [1*], Yue Zhu [1], Hui Yan [1], Da-wei Qian [1], Han-qing Wang [2], Jian-qiangYu [2], Jin-ao Duan [2*]

[1] Jiangsu Collaborative Innovation Center of Chinese Medicinal Resources Industrialization, State Administration of Traditional Chinese Medicine Key Laboratory of Chinese Medicinal Resources Recycling Utilization, National and Local Collaborative Engineering Center of Chinese Medicinal Resources Industrialization and Formulae Innovative Medicine, Nanjing University of Chinese Medicine, Nanjing 210023, China

[2] School of Pharmacy, Ningxia Medical University, Yinchuan 750021, China

* Author to whom correspondence should be addressed: Sheng Guo, guosheng@njucm.edu.cn.; Tel.: +86 25 8581 1916, Jin-ao Duan, dja@njucm.edu.cn.; Tel: +86 25 8581 1291;

1. Supplementary Methods (Validation of the UPLC-TQ-MS/MS method)

The method was validated for linearity, limits of detection and quantification (LODs and LOQs), precision (inter-day and intra-day precision), repeatability, stability and accuracy following the International Conference on Harmonization (ICH) guideline.

1.1 Calibration Curves, LODs, and LOQs

Mixed standard stock solution containing the reference compounds of protochatechuic acid, caffeic acid, vanillic acid, rutin, calycosin-7-*O*-β-D-glucoside, hyperoside, ferulic acid, astragalin, isorhamnetin-3-*O*-β-D-glucoside, (-)-methylinissolin-3-*O*-β-D-glucoside, quercetin, calycosin, kaempferol, isorhamnetin, formononetin, rhamnocitrin was prepared in 90% methanol, and the concentration for the 16 analytes were as follows: 0.432, 0.049, 0.049, 0.286, 0.238, 0.049, 0.354, 0.180, 0.223, 0.291, 0.320, 0.053, 0.049, 0.036, 0.461, and 0.272 mg/mL. Working standard solutions for calibration curves were prepared by diluting the mixed standard stock solution with 80% methanol to different concentrations.

The linearity was obtained by preparing at least six standard solutions of the appropriate concentration. Calibration curves were constructed from peak areas of the reference standards versus their concentrations. The limits of detection (LODs) and quantification (LOQs) for each analyte under the present chromatographic conditions were determined by diluting the standard solution when the signal-to-noise ratios (S/N) of analytes were about 3 and 10, respectively.

1.2 Precision, repeatability and stability

To evaluate the precision of the method, six replicate standard solutions were analyzed and the relative standard deviation (RSD) of the peak area of each standard compound was calculated. To verify the repeatability of the method, six different sample solutions were prepared from the same sample and the variations were represented by RSD. To investigate the stability of the sample, the sample solutions were stored at 4 ℃ and analyzed at different time points (0, 2, 4, 6, 8, 12, 16, and 24 h).

2. Supplementary figures

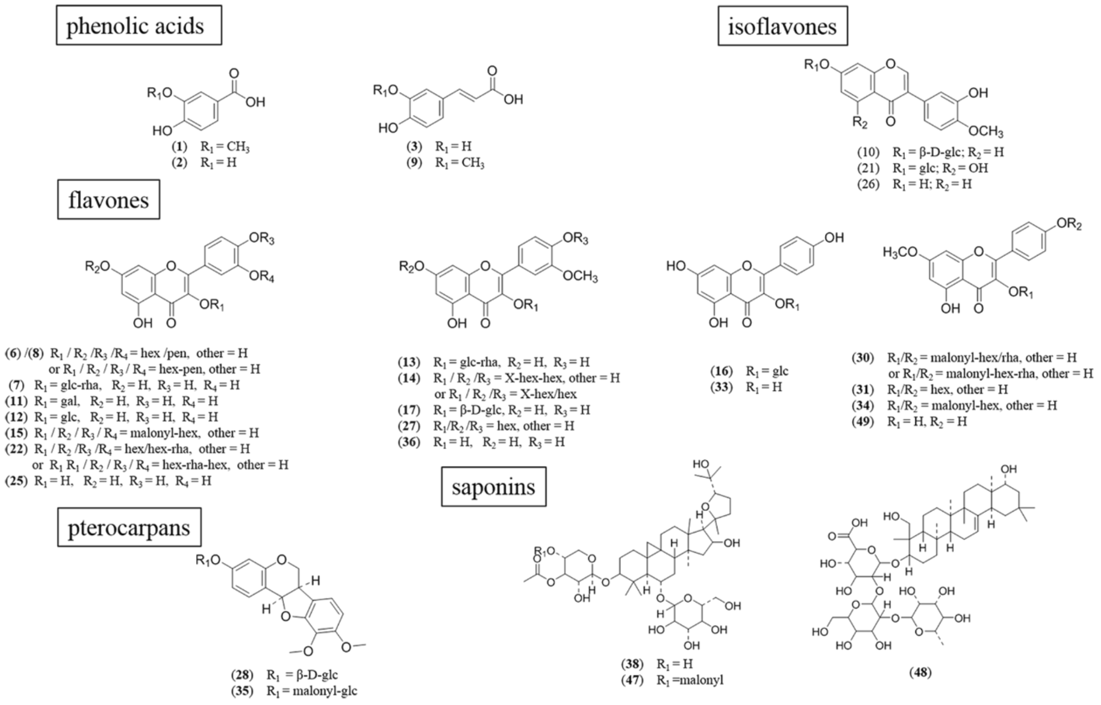

Figure S1. Structures of the 31 compounds identified in ME

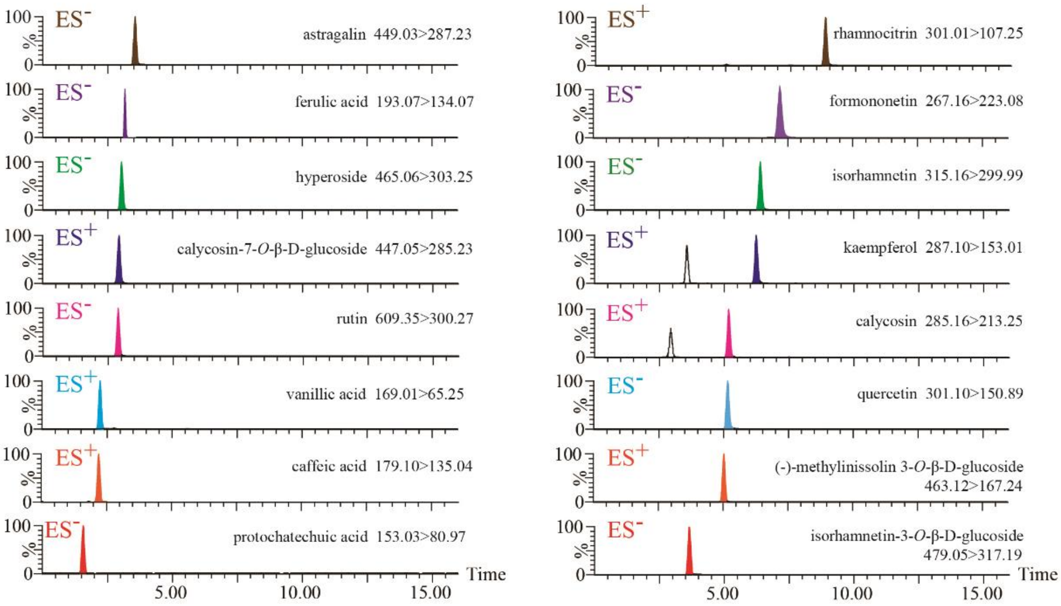

Figure S2 UPLC-TQ-MS/MS MRM chromatograms of mixture standards

3. Supplementary tables

Table S1. Calibration curves, LODs and LOQs of the 16 phenolic acids and flavonoids.

Analytes	Calibration Curves	R^2	Range (μg/mL)	LOD (ng/mL)	LOQ (ng/mL)
protochatechuic acid	$y=2.990\times10^5x+2.359\times10^2$	0.9980	0.42–54.00	1.550	4.649
caffeic acid	$y=8.625\times10^6x+4.068\times10^2$	0.9988	0.05–6.09	0.072	0.217
vanillic acid	$y=5.333\times10^6x-6.743$	0.9995	0.05–6.07	0.036	0.107
rutin	$y=7.166\times10^6x+7.682\times10^2$	0.9980	0.28–35.80	0.063	0.190
calycosin-7-*O*-β-D-glucoside	$y=2.931\times10^6x+9.086\times10$	0.9917	0.23–29.7	0.018	0.053
hyperoside	$y=3.929\times10^6x-2.588\times10^2$	0.9996	0.05–6.07	0.121	0.364
ferulic acid	$y=5.380\times10^6x+8.569\times10^2$	0.9993	0.35–44.30	0.009	0.026
astragalin	$y=4.956\times10^7x+6.496\times10^2$	0.9999	0.18–22.45	0.048	0.143
isorhamnetin-3-*O*-β-D-glucoside	$y=6.203\times10^7x-7.405\times10^2$	0.9993	0.22–27.91	0.018	0.054
(-)-methylinissolin-3-*O*-β-D-glucoside	$y=1.123\times10^7x+1.284\times10^2$	0.9950	0.28–36.41	0.020	0.059
quercetin	$y=3.360\times10^7x+7.270\times10$	0.9999	0.31–40.05	0.026	0.078
calycosin	$y=2.302\times10^7x+2.364\times10^3$	0.9957	0.05–6.67	0.086	0257
kaempferol	$y=2.573\times10^7x+7.421\times10^2$	0.9980	0.05–6.13	0.014	0.043
isorhamnetin	$y=1.053\times10^8x+1.240\times10^3$	0.9951	0.04–4.55	0.006	0.018
formononetin	$y=1.740\times10^8x+1.020\times10^4$	0.9958	0.45–57.65	0.001	0.002
rhamnocitrin	$y=3.682\times10^7x+1.291\times10^3$	0.9984	0.27–33.98	0.013	0.039

Table S2. Precision, repeatability, stability, and recovery of the 16 phenolic acids and flavonoids.

Analytes	Precision (%, n=6)		Repeatability (%, n=6)	Stability (%, n=6)	Recovery (%, n=3)	
	Intra-day	Inter-day			Mean	RSD
protochatechuic acid	3.08	4.28	4.70	1.63	101.31	4.73
caffeic acid	0.27	3.41	2.83	2.06	96.10	3.23
vanillic acid	2.45	3.07	2.80	3.86	102.39	3.93
rutin	3.42	4.10	1.29	2.80	100.23	3.52
calycosin-7-*O*-β-D-glucoside	3.37	3.94	3.16	1.32	99.03	4.03
hyperoside	1.27	2.22	0.68	2.41	101.92	2.64
ferulic acid	0.75	2.83	3.00	4.46	98.23	3.53
astragalin	1.35	3.25	0.57	2.63	101.08	3.13
isorhamnetin-3-*O*-β-D-glucoside	2.14	3.62	0.96	1.65	99.52	2.68
(-)-methylinissolin-3-*O*-β-D-glucoside	2.78	4.02	1.89	2.78	102.23	3.02
quercetin	3.08	4.11	4.15	3.59	99.20	4.69
calycosin	2.56	3.21	4.85	1.90	101.32	2.34
kaempferol	1.80	2.39	3.16	2.56	102.10	3.04
isorhamnetin	1.95	3.82	3.00	3.72	98.62	3.67
formononetin	2.10	3.21	2.95	1.93	95.31	4.20
rhamnocitrin	1.00	3.84	3.68	1.29	99.01	3.53

Table S3. MS/MS detection parameters of 16 phenolic acids and flavonoids in AMF extracts.

Analytes	Retention time (min)	MRM transitions	Cone voltage (V)	Collision energy (eV)
protochatechuic acid	1.56	153.03>80.97	24.0	16.0
caffeic acid	2.20	179.10>135.04	22.0	14.0
vanillic acid	2.24	169.01>65.25	18.0	14.0
rutin	2.87	609.35>300.27	52.0	34.0
calycosin-7-*O*-β-D-glucoside	2.94	447.05>285.23	22.0	20.0
hyperoside	3.03	465.06>303.25	16.0	14.0
ferulic acid	3.16	193.07>134.07	20.0	14.0
astragalin	3.56	449.03>287.23	14.0	10.0
isorhamnetin-3-*O*-β-D-glucoside	3.66	479.05>317.19	14.0	14.0
(-)-methylinissolin-3-*O*-β-D-glucoside	5.02	463.12>167.24	12.0	28.0
quercetin	5.15	301.10>150.89	36.0	22.0
calycosin	5.17	285.16>213.25	50.0	34.0
kaempferol	6.23	287.10>153.01	44.0	28.0
isorhamnetin	6.40	315.16>299.99	38.0	22.0
formononetin	7.21	267.16>223.08	44.0	32.0
rhamnocitrin	8.85	301.01>107.25	48.0	40.0
